# Thephining for Cerebral Hemorrhage

**Published:** 1892-02

**Authors:** Emory Lanphear

**Affiliations:** Kansas City, Mo., Surgeon East Side Free Dispensary, Professor of Orthopædic Surgery in the University Medical College


DANIEL'S

### TEXAS MEDICAL JOURNAL.

A Representative Organ of the Medical Profession, and an Exponent of Rational Medicine; devoted to the Organization, Advancement and Elevation of the Profession in Texas.

PUBLISHED MONTHLY.—SUBSCRIPTION $2.00 A YEAR.

Vol. VII. AUSTIN, FEBRUARY, 1892. No. 8.

### Original Articles.

CONTRIBUTED EXCLUSIVELY TO THIS JOURNAL.

*The Articles in this Department are accepted and published with the understanding that we are not responsible for, nor do we indorse the views and opinions of the writers by so doing.*

For Daniel's Texas Medical Journal.

### A CASE OF SUCCESSFUL TREPHINING FOR CEREBRAL HEMORRHAGE.

BY EMORY LANPHEAR, M. D., PH. D., KANSAS CITY, MO.,
Surgeon East Side Free Dispensary, Professor of Orthopædic Surgery in the University Medical College.

DR. W. C. B., age 47, had a slight attack of cerebral hemorrhage in the early part of 1891. I saw him at Carthage, Mo., October 10, 1891, in consultation with Dr. R. T. Scott. The following is the entry in my case book: "Patient had stroke of paralysis about two weeks ago; had all the symptoms of cerebral hemorrhage, has not entirely recovered consciousness since; has right hemiphlegia and a complete aphasia. Has been rapidly emaciating; has lately had paralysis of sphincters, dysphagia and fever (temperature 100° to 101° F.); strength rapidly failing—to an alarming degree past few days; indicates that he has terrible pain and tightness in head, with insomnia therefrom.

At 3 p. m., assisted by Drs. Mathews, Scott, King and Schafer,

I opened the skull over motor and speech centers; no pulsation of dura; on opening dura found some softening of arm center; enlarged fenestrum and examined speech center; no evidence of clot there, but indications of severe pressure. Removed remnants of clot (sub-cortical) and some of softened arm center, irrigated with hot bichloride solution, 1-5000, put in cat-gut drainage and closed. Duration 1 hour and 35 minutes. Put to bed in good shape, with little shock. Pulsation of brain returned (at opening in skull) after operation.

"October 11, slept finely all night on morphine (gr. ¼) and atropine (gr. 1-60). At 9 a. m. is feeling first-rate, better than at any previous time; mind much improved; can say, 'O, yes,' quite distinctly, and can move foot to a marked extent. At 4 p. m. dressed wound and removed drainage; applied large quantity of iodoform and put on permanent dressing."

The subsequeut history is given by Dr. Scott thus: "Three hours after our redressing the wound the temperature dropped from 100½° to 99 3-5°. On the following day (Oct. 13) temperature 99°, pulse 110, stomach irritable; applied mustard and ordered milk and lime water in small quantities, frequently repeated. At 4 p. m. temperature 100½°, pulse 110 and intermittent; patient restless; gave sulphonal, half scruple, in milk, repeated in two hours; also, hydrargyri protoiodid., gr. ¼, every two hours until bowels moved. October 14, 9 a. m. temperature normal, pulse 100, and regular; appetite good; can urinate without catheter. At 7 p. m. temperature normal; bowels acted freely from mercury; having slept well the night before, sulphonal was repeated. October 15, condition improving. October 20, on removing dressings found the head as dry and clean as when first applied; wound healed by immediate union. Patient is able to sit up several hours each day and is entirely free from headache he had prior to operation."

The patient came to Kansas City (203 miles from Carthage), on October 30. The amount of improvement was so great as to actually shock me. And a letter written by Dr. Scott, Nov. 10, says, "Dr. B. is still improving."

Macewen, of Glasgow, trephined a patient for cerebral hemorrhage as early as 1883, successfully, and since that date there have been about thirty-five operations for this tronble. In view of the fact that the lesion can usually be located so accurately, is commonly so accessible, and the results of internal medication so unsatisfactory, it is, indeed, a strange thing that craniectomy is not more frequently made. Especially in the ingravescent form of cerebral hemorrhage (termed "progressive apoplexy" by some authors) should operation be successful if done before the blood breaks into the ventricles. I am aware that most authorities agree in condemning the attempt at removal of the clot, but I am still of the opinion expressed before a class of the University of Kansas City, in 1886, that the time will come when the surgeon will operate for bleeding beneath the arachnoid, in the cortex and in the white matter as well—in fact, in any case where the ruptured vessel is not within the internal capsule.

If one is familiar with the technique of cerebral surgery there need be no fear of doing serious mischief. Lucas Champonierre has collected the histories of thirty cases without a death or an untoward circumstance. If my own record can be taken as a guide as to danger in operative procedures in the brain in cases not traumatic in origin, cerebral surgery is fully as safe as abdominal. I have operated twenty-three times (including this case of appoplexy) for tumor, abscess, imbecility, etc., with only two deaths—a mortality far less than that found in the early years of laparotomy.

The time for operating is of course a question of importance. If it were possible to operate at the time of the "stroke," and see the still bleeding vessel, as I have done in one case of rupture of the middle meningeal artery, removing at the same time the clot already formed, that would be the ideal time for selection. But this is entirely out of the question in most cases, and it is only after the lapse of several days that one can tell if the attack is going to be fatal. So upon general principles I would say, that if the paralysis (or aphasia) has not begun to improve markedly after the eighth day, operation may be advised. On the other hand remarkable results may be obtained by opening the skull and cleaning out the remnants of the clot even weeks after the initial lesion. If red softening has not taken place, if tissues necrosis have not occurred from pressure, and if there have been no very extensive laceration of the corona radiata, restoration may result after operation in cases of apparently hopeless hemiplegia.

